# Intrinsic stabilization of vacancies in catalysts *via* high-entropy approach for lithium-sulfur batteries

**DOI:** 10.1093/nsr/nwaf375

**Published:** 2025-09-08

**Authors:** Chenghao Zhao, Yang Huang, Pengyu Wang, Zhaoyu Chen, Yu Zhang, Naiqing Zhang

**Affiliations:** State Key Laboratory of Urban-rural Water Resource and Environment, School of Chemistry and Chemical Engineering, Harbin Institute of Technology, Harbin 150001, China; Qingdao New Energy Shandong Laboratory, Qingdao Institute of Bioenergy and Bioprocess Technology, Chinese Academy of Sciences, Qingdao 266101, China; State Key Laboratory of Urban-rural Water Resource and Environment, School of Chemistry and Chemical Engineering, Harbin Institute of Technology, Harbin 150001, China; State Key Laboratory of Urban-rural Water Resource and Environment, School of Chemistry and Chemical Engineering, Harbin Institute of Technology, Harbin 150001, China; Space Environment Simulation Research Infrastructure, Harbin Institute of Technology, Harbin 150006, China; Department of Chemistry, Stockholm University, Stockholm 10691, Sweden; State Key Laboratory of Urban-rural Water Resource and Environment, School of Chemistry and Chemical Engineering, Harbin Institute of Technology, Harbin 150001, China

**Keywords:** lithium sulfur batteries, catalysts, stability, oxygen vacancies, high-entropy oxide

## Abstract

Oxygen vacancies (V_O_) have been considered as a significant strategy to improve the performance of catalysts in Li-S batteries. However, the highly active V_O_ are a double-edged sword, as their instability can undermine long-term cycle performance. Therefore, it is essential to stabilize V_O_ while maintaining their high activity. Here, five different metal elements are dissolved into the lattice structure of two-dimensional oxides to construct intrinsically stable and active V_O_ for better lithium-sulfur catalysts. The unique electronic and crystal structure in high-entropy oxide endows the changed differentiated formation energies and high diffusion energy barrier of V_O_ to form intrinsically stable V_O_. The Li-S batteries with stable V_O_ in the electrocatalyst deliver a high specific capacity of 1301 mAh g^−1^ at 0.2C and low capacity fading of 0.032% per cycle after 2000 cycles at 1C. This work will inspire efforts on breaking the trade-off between activity and stability in heterogeneous catalysis beyond Li-S batteries.

## Teaser

This study creates intrinsically stable and highly active catalytic sites by constructing a 2D high-entropy oxide material, leading to lithium-sulfur batteries with exceptional capacity and stability.

## INTRODUCTION

Most countries in the world have taken it as an important strategic target to reduce dependence on fossil fuels and vigorously develop new sources of renewable energy. Lithium-sulfur batteries (LSBs), with their remarkable energy density and low cost compared with traditional lithium-ion batteries, will play a pivotal role in the transformation of the future energy storage market [1–3]. Unfortunately, the sluggish reaction kinetics and serious shuttle effect of lithium polysulfides (LiPSs) in sulfur cathodes significantly reduce the energy density and cycle stability of LSBs [1–6].

In recent years, various electrocatalysts have been proposed in LSBs to anchor LiPSs and accelerate reaction kinetics through rational design and construction [7–9]. Introducing vacancies into the catalyst is proving to be a powerful strategy in boosting catalytic performance towards LiPSs conversion in LSBs. Compared to other defect types, oxygen vacancies (V_O_) can be more readily introduced into materials [10]. They expose a greater number of under-coordinated atoms, optimize the electronic structure, and serve as active sites for adsorbing and catalyzing LiPSs. Furthermore, V_O_ reduce the bandgap and enhance electrical conductivity [11]. Consequently, catalytic materials with V_O_ have been extensively investigated and applied in LSBs [12,13]. Typically, V_O_ on the surface of catalytic materials can act as highly active sites for adsorption and catalysis of LiPSs [14–16]. For example, Li *et al.* introduced V_O_ onto the surface of TiO_2_ by calcining, which significantly improved electrical conductivity and catalytic performance [17]. Zhu *et al.* introduced rational V_O_ into Mn_3_O_4_ by surface engineering, which accelerated the conversion of LiPSs and suppressed the ‘shuttle effect’ [18]. However, these highly active vacancies bring conflicts in maintaining its stability during repeated discharge/charge cycles, resulting in decreased catalytic performance upon cycling [19–21]. Besides, V_O_ constructed on the surface cannot also be enriched under the concentration gradient, thus failing to guarantee abundant active sites [22–24]. The holistic change of intrinsic properties is a more powerful approach than postprocessing methods such as doping and constructing barrier layers [25,26]. Therefore, there is an urgent need for developing strategies to build intrinsically stable surface V_O_ while maintaining their high activity.

High-entropy oxide (HEO), characterized by the incorporation of five or more distinct elements into an oxide lattice, exhibits a series of unique properties including the cocktail effect and the slow diffusion effect [27,28]. This is primarily due to the homogeneous dispersal of elements with disparate properties within the lattice [29–32]. In addressing the challenge of V_O_, the multielement solid solution structure is expected to disrupt the stable diffusion pathways of V_O_, thereby enriching and stabilizing the surface V_O_. This highlights the potential of a high-entropy strategy to simultaneously achieve highly active and intrinsically stable V_O_ on the surface of catalysts.

Herein, five different metal elements were dissolved into the lattice structure of 2D CeO_2_ to construct intrinsically stable and active V_O_ for LSBs with high performance. Experiments and density functional theory (DFT) calculations show that the underlying mechanism for stabilizing surface V_O_ is attributed to the differentiated formation energies and high diffusion energy barrier of V_O_, endowed by the unique electronic and crystal structure in the high-entropy system. The assembled LSBs, incorporating electrocatalysts with V_O_ stabilized by a high-entropy strategy, deliver a specific capacity as high as 1301 mAh g^−1^ under 0.2C and a low average capacity fading of 0.032% per cycle after 2000 cycles under 1C. Besides, an areal capacity of 7.58 mAh cm^−2^ is exhibited under 0.2C at a high sulfur areal loading of 6.4 mg cm^−2^.

## RESULTS AND DISCUSSION

### Synthesis and characterization of materials

**Figure 1.**
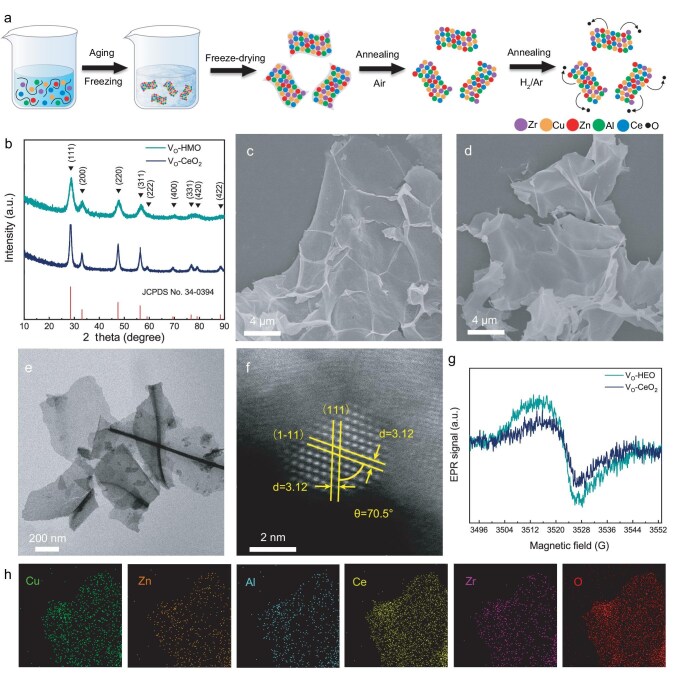
(a) Schematic diagram of the synthesis of V_O_-HEO. (b) XRD patterns. SEM images of (c) V_O_-HEO and (d) V_O_-CeO_2_**.** (e) TEM image and (f) AC-STEM image of V_O_-HEO. (g) EPR spectrum of V_O_-HEO and V_O_-CeO_2_. (h) Elemental mapping images of V_O_-HEO.

Two-dimensional (2D) HEO can be obtained by a simple template synthesis [33]. A schematic diagram of this synthesis is shown in Fig. 1a. Five different metal ions (Ce, Cu, Al, Zn and Zr) are mixed with PVP in an aqueous solution. PVP is self-assembled into 2D micelles with five different metal ions adsorbed on the surface. Two-dimensional PVP powders loaded with metals are obtained by direct freeze-drying. The PVP template is removed after annealing in air, and the mixed metal ions form 2D HEO. Finally, more oxygen atoms are removed from the surface in a reducing atmosphere to obtain HEO with surface V_O_ (V_O_-HEO). Each peak in the XRD pattern of V_O_-HEO corresponds to the diffraction peaks from JCPDS No. 34–0394 (Fig. 1b), with no additional peaks appearing. The XRD pattern indicates that the five metal ions are solidly dissolved into a complete face-centered cubic (FCC) structure. In addition, we synthesized material with the same FCC structure to explore the effect of high entropy. Ce is added in the first step to obtain CeO_2_ with surface V_O_ (V_O_-CeO_2_). V_O_-CeO_2_ has the same FCC structure as V_O_-HEO. It can be clearly observed through scanning electron microscopy (SEM) (Fig. 1c and d) that V_O_-HEO and V_O_-CeO_2_ have a similar 2D morphology. Transmission electron microscope (TEM) imaging of V_O_-HEO shows that the synthesized samples have a 2D morphology (Fig. 1e). The high-entropy material was characterized by spherical aberration corrected scanning transmission electron microscopy (AC-STEM) to directly observe the lattice structure (Fig. S1). Figure 1f is obtained by zooming in on the area numbered 1 in Fig. S1, where the lattice planes at the atomic level can be clearly observed. The two planes with a spacing of 3.12 Å have an angle of 70.5°, corresponding to the (111) and (1–11) lattice planes in the FCC structure, respectively.

Electron paramagnetic resonance (EPR) confirms that V_O_ are successfully introduced into the materials (Fig. 1g). V_O_-HEO has a higher concentration of V_O_ owing to more unsaturated atoms. Furthermore, high-angle annular dark-field-scanning transmission electron microscope (HAADF-STEM) images and corresponding elemental mapping images show that Ce, Cu, Zn, Al, Zr and O are uniformly distributed on the 2D nanosheets, which is the fingerprint feature of high-entropy materials (Fig. 1h) [34,35]. The elemental mapping images of V_O_-CeO_2_ show that there are no heteroatoms in V_O_-CeO_2_ (Figs S2 and S3).

### Mechanism of stabilizing V_O_

**Figure 2.**
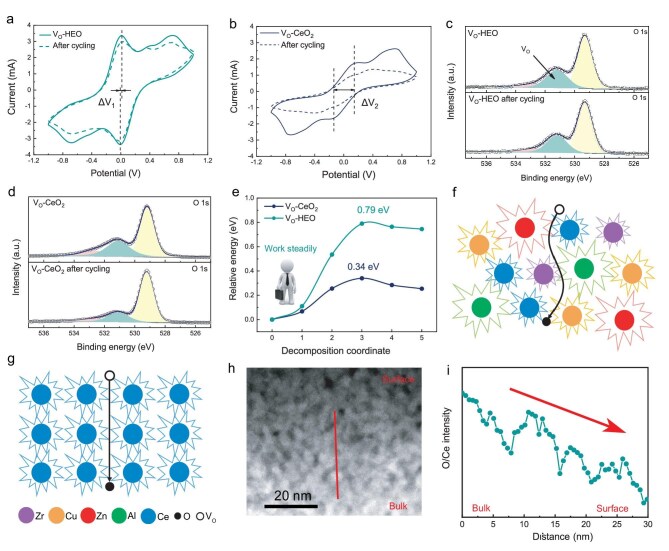
CV curves of the symmetric cells with (a) V_O_-HEO and (b) V_O_-CeO_2_ electrodes at different cycle numbers. (c, d) O1s XPS comparative analysis before and after CV cycling of the symmetric cells. (e) Diffusion barrier of V_O_ in V_O_-HEO and V_O_-CeO_2_. Schematic diagram of V_O_ inward diffusing of (f) V_O_-HEO and (g) V_O_-CeO_2_. (h–i) AC-STEM image and EELS of V_O_-HEO.

The cyclic voltammetry (CV) test of Li_2_S_6_ symmetric cells is an important method to evaluate the performance of catalysts [36]. Long-cycle CV tests were performed to compare the catalytic activity and stability of V_O_-HEO and V_O_-CeO_2_ (Fig. 2a and b). The smaller distance between symmetric peaks (ΔV_1_<ΔV_2_) reflects the lower polarization. The higher peak value and larger peak area of V_O_-HEO indicate better catalytic performance [37]; the peak value and peak area of V_O_-CeO_2_ decrease significantly after 100 cycles. In contrast, V_O_-HEO can still maintain its CV curve and shows better catalytic stability. To explore the reasons for the decrease in catalytic performance, the elemental chemical states of the catalyst’s surface were tested by X-ray photoelectron spectroscopy (XPS) (Fig. 2c and d, Fig. S4). The characteristic peak of V_O_ can be observed in the O1s high resolution XPS of V_O_-HEO and V_O_-CeO_2_, indicating the existence of surface V_O_ [38]. The concentration of V_O_ on the V_O_-CeO_2_ surface decreases significantly after cycling compared with the stable V_O_ on V_O_-HEO. Since the catalytic reaction occurs on the surface, this makes it difficult for the V_O_ in the catalyst bulk to participate in the reaction as active sites. Therefore, the decreased concentration of surface V_O_ leads to the loss of active sites, which explains the degradation of catalytic performance.

The mechanism of stabilizing surface V_O_ in HEO was simulated and analyzed by DFT calculation. V_O_-HEO has obvious lattice distortion compared with V_O_-CeO_2_ in the optimized models (Fig. S5). This is because the five metals with different coordination states and atomic radii are confined to the same lattice [39]. Atoms are rearranged, causing lattice distortion [40]. The inward diffusion of V_O_ was simulated by climbing image nudged elastic band (CI-NEB). The calculation results show that V_O_-HEO has a higher energy barrier of V_O_ inward diffusion (0.79 eV) than V_O_-CeO_2_ (0.34 eV) (Fig. 2e). By observing the local atomic states of the diffusion process (Fig. S6), it is found that the atomic spacing in Ce-Ce and Al-Zr is 3.87 Å and 3.26 Å, respectively. The distortion of atomic spacing leads to the difficult diffusion of O atoms [41]. The energy difference between the beginning and end states of the inward diffusion process in V_O_-HEO is larger than that in V_O_-CeO_2_, indicating that the inward diffusion of V_O_ in V_O_-HEO is more thermodynamically difficult. The calculated results of formation energies of V_O_ at different positions are shown in Fig. S7. The formation energies of V_O_ are very different because of the distinct local environments of surface and internal V_O_ in V_O_-HEO [39]. The V_O_ are more likely to exist on the surface owing to lower formation energies [42,43]. Due to the various coordination environments and charge states of the five metal atoms, the local energies of sites are significantly different [44]. As a result, coordination atoms and different neighbor atoms at each lattice site induce trapping effects and impede the diffusion process when O atoms jump into vacancies [45]. Different sites with diverse local energy and lattice distortions provide complex and rugged paths, which act as a shield against the inward diffusion of V_O_ (Fig. 2f and g). On the contrary, the local environments of V_O_ in V_O_-CeO_2_ are similar. There is little difference in formation energies of V_O_, which leads to easier diffusion. Besides, V_O_-HEO has lower formation energy than V_O_-CeO_2_, which is conducive to form V_O_ [46–48].

AC-STEM was used to directly observe the distribution of V_O_ in V_O_-HEO to demonstrate the role of the high-entropy effect in stabilizing surface V_O_. Figure 2h is obtained by zooming in on the area numbered 2 in Fig. S1. This area is viewed from the side of the 2D materials where the vertical distribution of V_O_ can be observed. The contrast in HAADF mode is proportional to the average atomic number [49]. The contrast gradually deepens from bulk to surface, indicating that the V_O_ are enriched on the surface. Electron energy loss spectroscopy (EELS) can be used to further confirm the distribution of V_O_. Using Ce as a calibration, the O/Ce ratio of V_O_-HEO is measured by EELS line scanning from bulk to surface (Fig. 2i). The results show a decrease of the O/Ce ratio indicating that the oxygen vacancy concentration increases from bulk to surface.

In summary, the high-entropy strategy changes the intrinsic properties due to lattice distortion and differences of local states, increases the diffusion energy barrier of the oxygen vacancy, and forms the intrinsically stable V_O_ on the surface. Therefore, V_O_-HEO shows a more stable catalytic performance.

### Analysis of anchoring capacity

**Figure 3.**
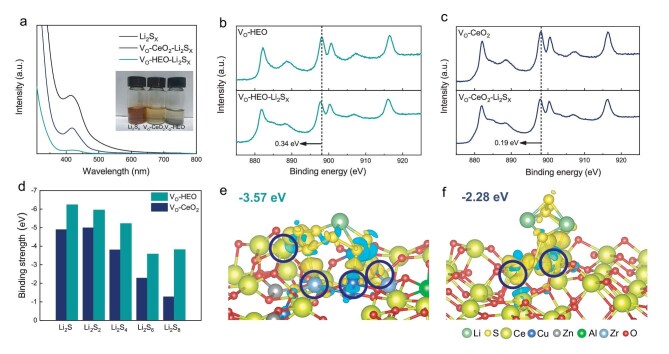
(a) UV-vis spectra and corresponding optical photograph of the Li_2_S_X_ solutions adsorbed by V_O_-HEO and V_O_-CeO_2_ for 10 h. Ce 3d XPS comparative analysis of (b) V_O_-HEO and (c) V_O_-CeO_2_ before and after adsorption of Li_2_S_X_. (e) Binding strength of different sulfur species adsorbed on V_O_-HEO and V_O_-CeO_2_. Differential charge diagram of (e) V_O_-HEO and (f) V_O_-CeO_2_ after adsorption of Li_2_S_6_.

A visual adsorption experiment can be used to understand the anchoring capacity of the catalysts for LiPSs (Fig. 3a). The original Li_2_S_X_ solution has an orange-yellow color. The Li_2_S_X_ solution becomes almost colorless after adsorption by V_O_-HEO, compared to the bright yellow color of V_O_-CeO_2_. XPS was employed to analyze the catalysts after adsorption of Li_2_S_X_. The shift of Ce 3d in V_O_-HEO is greater after adsorption (0.34 eV) compared with the V_O_-CeO_2_ (0.19 eV) (Fig. 3b and c) [50], suggesting a greater interaction between V_O_-HEO and LiPSs. To further understand the adsorption mechanism of V_O_-HEO, DFT calculations were employed to simulate the adsorption of various LiPSs on the surfaces of V_O_-HEO and V_O_-CeO_2_. The binding energies between V_O_-HEO and LiPSs are negative compared to that of V_O_-CeO_2_ (Fig. 3d). The charge density difference analysis of the adsorption system was calculated for a more intuitive understanding of the adsorption mechanism of V_O_-HEO (Fig. 3e and f). The yellow and blue parts represent the accumulation and loss of electrons, respectively. The locations circled are the sites where the catalysts bind to LiPSs. The surface V_O_ of V_O_-HEO exposes a variety of metal atoms, which binds to LiPSs as adsorption sites. In contrast, V_O_-CeO_2_ has only a few adsorption sites. There are larger volumes of yellow and blue parts formed between Li_2_S_6_ and V_O_-HEO, indicating that V_O_-HEO has stronger adsorption sites. The binding energy between V_O_-HEO and Li_2_S_6_ (−3.57 eV) is more negative than that of V_O_-CeO_2_ (−2.28 eV). In summary, the surface V_O_ of V_O_-HEO expose more unsaturated metal atoms as adsorption sites, which is conducive to the adsorption of S atoms in LiPSs [51,52]. The resulting increased anchoring capacity to LiPSs can effectively inhibit the shuttle effect [53].

### Analysis of catalytic performance

**Figure 4.**
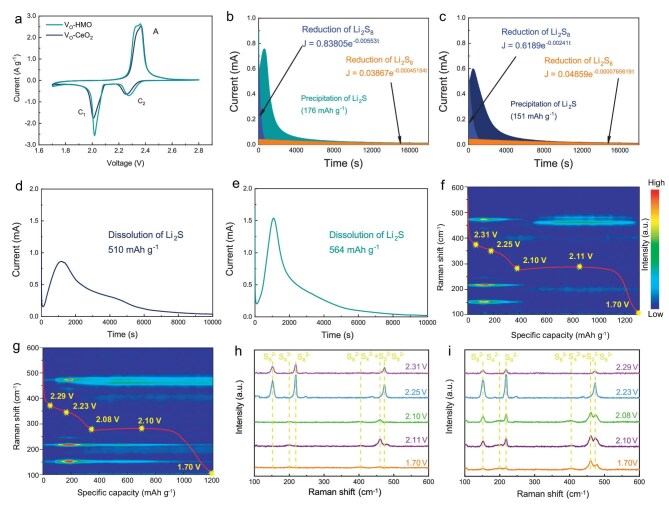
(a) CV curves. Potentiostatic discharge profiles of Li_2_S nucleation on (b) V_O_-HEO and (c) V_O_-CeO_2_. Potentiostatic charge profiles of Li_2_S decomposition on (d) V_O_-CeO_2_ and (e) V_O_-HEO. *In situ* Raman contour plots and corresponding discharging curves of (f) V_O_-HEO and (g)V_O_-CeO_2_. *In situ* Raman spectra of (h) V_O_-HEO and (i) V_O_-CeO_2_ at different voltages.

To investigate the effect of HEO on the dynamics of LSBs, V_O_-HEO and V_O_-CeO_2_ were coated onto a commercial polypropylene separator. The digital photos show that the catalysts are uniformly modified on the separators (Fig. S8). The bending test and SEM images of the interface show that the catalysts combine tightly with separators. These separators coated with different materials are presented in Fig. S9, where the V_O_-HEO and V_O_-CeO_2_ functional materials exhibit 2D morphologies similar to the previous text (Fig. S9a and c). Conductive carbon particles are observed to be sandwiched between these 2D architectures. Low-magnification SEM characterization further confirms the homogeneous distribution of both V_O_-HEO/V_O_-CeO_2_ functional materials and conductive carbon components across the separator surfaces (Fig. S9b and d). The modified separators were assembled into LSBs and CV measurements were carried out with a scan rate of 0.1 mV s^−1^ (Fig. 4a). The obtained CV curve can be divided into one anodic peak (A) and two cathodic peaks (C1 and C2). The cathodic peaks and anodic peak correspond to the sulfur reduction reaction during discharge and sulfur oxidation reaction during charge, respectively [54,55]. V_O_-HEO based cells show obvious positive shift of peaks C1 and C2 and negative shift of peak A, indicating lower polarization than V_O_-CeO_2_ based cells. The V_O_-HEO based cells have lower Tafel slopes corresponding to C1, C2 and A, indicating faster reaction kinetics than V_O_-CeO_2_ based cells (Fig. S10), respectively. The Li_2_S nucleation of soluble short-chain LiPSs into solid Li_2_S contributes a large capacity to the discharge process. The effects of catalysts on the liquid-solid phase conversion reaction can be analyzed by a Li_2_S nucleation experiment. Li_2_S_8_ was used to assemble cells, and the Li_2_S nucleation curve was obtained by discharging potentiostatically (Fig. 4b and c). Li_2_S nucleation curves were fitted to separate the capacity contributed by other processes. V_O_-HEO based cells have a higher current peak and a larger capacity contributed by Li_2_S nucleation (176 mAh g^−1^) compared to V_O_-CeO_2_ based cells (151 mAh g^−1^). The process of Li_2_S decomposition was studied by charging potentiostatically after completing discharge (Fig. 4d and e). V_O_-HEO based cells have a higher current peak and a larger capacity contributed by Li_2_S decomposition (564 mAh g^−1^) compared with V_O_-CeO_2_ based cells (510 mAh g^−1^). V_O_-HEO can effectively promote the Li_2_S nucleation and Li_2_S decomposition, thus accelerating the reaction kinetics of LSBs [56,57].

V_O_-HEO and V_O_-CeO_2_ based cells were tested by *in situ* Raman spectroscopy to further understand the catalysis process (Fig. 4f–i) [58]. The different corresponding characteristic peaks of LiPSs are shown in Table S1. In *in situ* Raman spectroscopy, the characteristic peaks of 156, 217, and 475 cm^−1^ correspond to Li_2_S_8_ [59,60]. The characteristic peak of 393 cm^−1^ corresponds to Li_2_S_6_ while the characteristic peak of 201 cm^−1^ corresponds to Li_2_S_4_. The characteristic peak of 459 cm^−1^ corresponds to both Li_2_S_4_ and Li_2_S_2_ [60]. *In situ* Raman contour plots show the changes of LiPSs during the whole process (Fig. 4f and g). The existence of Li_2_S_8_ during the whole discharge process of V_O_-CeO_2_ based cells indicates a severe shuttle effect. In contrast, the Li_2_S_8_ of V_O_-HEO based cells are fully converted after the first discharge plateau. In addition, the conversion of LiPSs in different stages of V_O_-CeO_2_ based cells are delayed compared to V_O_-HEO based cells, indicating that V_O_-HEO can catalyze the conversion of LiPSs more effectively. The state of LiPSs in key stages during discharging are observed (Fig. 4h and i). First, the intensity of Li_2_S_8_ peaks increase rapidly during the first discharge plateau in V_O_-HEO based cells, indicating that the concentration of Li_2_S_8_ continues to increase as the solid sulfur is converted to Li_2_S_8_ (2.31 V and 2.25 V). Second, the intensity of Li_2_S_8_ peaks gradually weaken and disappear before the second plateau (2.10 V), and there are obvious Li_2_S_6_ and Li_2_S_4_ peaks. Then, there are only obvious Li_2_S_4_ and Li_2_S_2_ peaks (2.11 V), indicating that all long-chain LiPSs are converted into short-chain LiPSs. Finally, the Li_2_S_4_ and Li_2_S_2_ peaks disappear at the end of discharge, indicating that LiPSs are completely converted to Li_2_S (1.70 V). In contrast, the conversion of Li_2_S_8_ in V_O_-CeO_2_ based cells is slower (2.29 V and 2.23 V). After that, the Li_2_S_8_ peaks do not completely disappear and are not fully converted during the second plateau (2.08 V and 2.10 V). Finally, the Li_2_S_8_ peaks disappear but there is still a large amount of Li_2_S_4_ and Li_2_S_2_ that are not fully converted. The results of *in situ* Raman spectroscopy show that V_O_-HEO can achieve faster and more complete conversion of LiPSs and significantly inhibit the shuttle effect compared with V_O_-CeO_2_ [61,62].

**Figure 5.**
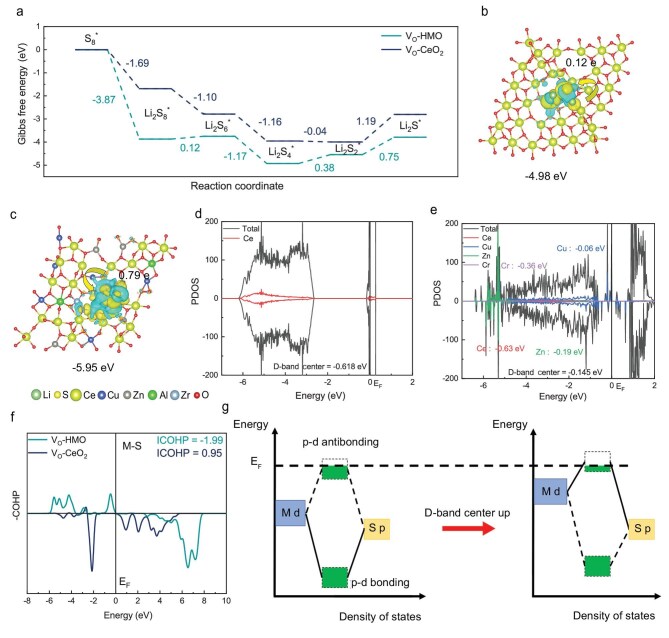
(a) Gibbs free energy changes of sulfur reduction processes on V_O_-HEO and V_O_-CeO_2_. The Bader charge numbers of atoms and differential charge diagrams of (b) V_O_-CeO_2_ and (c) V_O_-HEO after adsorption of Li_2_S_2_. PDOS of (d) V_O_-CeO_2_ and (e) V_O_-HEO. (f) COHPs of M-S bonds adsorbed on V_O_-HEO and V_O_-CeO_2_. (g) Schematic diagram of mechanism.

The discharge process is further simulated by DFT calculation. The Gibbs free energies (ΔG) for different steps of the discharge were obtained (Fig. 5a). The simulation results of different sulfur species are illustrated in Figs S11 and S12. During the reaction of *S_8_→*Li_2_S_8_→*Li_2_S_6_→*Li_2_S_4_→*Li_2_S_2_→ *Li_2_S, ΔG of the V_O_-CeO_2_ are −1.69 eV, −1.10 eV, −1.16 eV, −0.04 eV and 1.19 eV, whereas the ΔG of the V_O_-HEO are −3.87 eV, −0.12 eV, −1.76 eV, 0.38 eV and 0.75 eV. Li_2_S_2_→*Li_2_S is the rate-determining step (RDS) of the discharge process. V_O_-HEO has a lower energy barrier of RDS (0.75 eV) compared with V_O_-CeO_2_ (1.19 eV). V_O_-HEO is more conducive to catalyze the conversion of LiPSs during discharging [63–65]. To elucidate the catalytic mechanism of V_O_-HEO in reducing the reaction energy barrier of the RDS, differential charge analysis was performed on the adsorption process of the key intermediate Li_2_S_2_ (Fig. 5b and c). The results reveal that compared to V_O_-CeO_2_, the V_O_-HEO/Li_2_S_2_ system exhibits larger yellow and green iso-surfaces (representing electron accumulation and depletion, respectively), indicating enhanced charge transfer between V_O_-HEO and Li_2_S_2_ [66]. Bader charge analysis quantitatively verifies this observation: V_O_-HEO transfers 0.79 e to Li_2_S_2_, which is significantly higher than the 0.12 e transferred by V_O_-CeO_2_, demonstrating the superior charge exchange capability of V_O_-HEO [67]. To further investigate the origin of this enhanced charge transfer, projected density of states (PDOS) calculations and d-band center analyses were conducted (Fig. 5d and e). The d-band center of V_O_-HEO is elevated (−0.618 eV for V_O_-CeO_2_ vs −0.19 eV/−0.36 eV/−0.06 eV for Cu/Zn/Cr in V_O_-HEO), with Cu, Zn and Cr identified as primary contributors to d-band center upshifting, while non-d-orbital Al stabilizes the material structure. Crystal orbital Hamiltonian population (COHP) analysis of the metal-sulfur (M-S) bonds formed during Li_2_S_2_ adsorption shows that the anti-bonding states (negative COHP) and bonding states (positive COHP) in V_O_-HEO shift to higher energy levels (Fig. 5f). The integrated COHP (ICOHP) values quantify bond strength: V_O_-HEO exhibits a lower ICOHP (−1.99) compared to V_O_-CeO_2_ (0.95), indicating stronger bonding due to reduced anti-bonding occupancy below the Fermi level (E_f_) [68]. The mechanistic diagram (Fig. 5g) illustrates that the elevated d-band center in V_O_-HEO raises both p-d bonding and anti-bonding orbitals. Partial anti-bonding orbitals are pushed above E_f_, enabling preferential electron occupation in bonding orbitals (green region). This optimized orbital interaction enhances charge exchange efficiency, thereby strengthening catalyst-adsorbate interactions and improving catalytic performance toward LiPSs conversion [69,70].

The COHP of the adsorbed Li_2_S on catalysts was calculated to analyze the bonding state of the Li-S bonds (Fig. S13). The parts of −COHP >0 are the bonding states, and the parts <0 are the antibonding states. The energy of the Li-S bonding states on V_O_-HEO is higher compared with V_O_-CeO_2_, and some antibonding states appear below the Fermi level. In addition, the ICOHP of the Li-S bond on the HEO surface is more positive (−1.17 eV), reflecting a lower degree of bonding. The Li-S bond on the V_O_-HEO (2.70 Å) is longer than that on V_O_-CeO_2_ (2.32 Å) as can be seen through directly observing the length of the Li-S bonds in simulated Li_2_S (Fig. S14). V_O_-HEO is more beneficial to catalyze the Li_2_S decomposition during charging [54]. In summary, we have demonstrated both experimentally and theoretically that V_O_-HEO has better catalytic properties for LiPSs conversion during charging and discharging than V_O_-CeO_2_. V_O_-HEO exerts the unique cocktail effect through the synergistic interaction between various elements, which can effectively accelerate the reaction kinetics of LSBs [71,72].

### Battery performance

**Figure 6.**
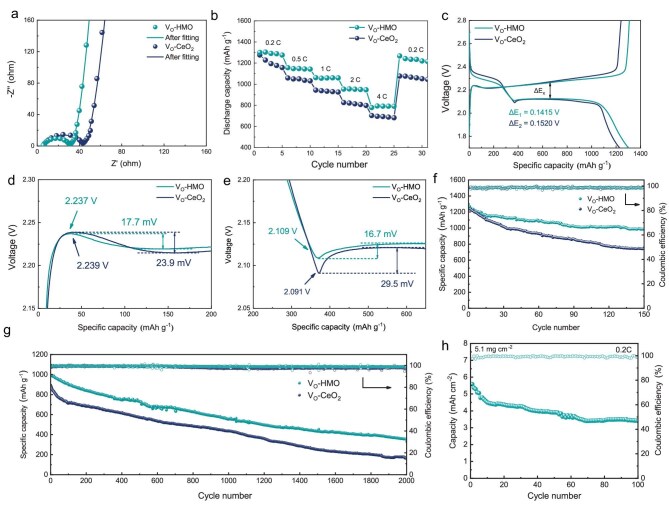
(a) The well-fitted Nyquist plots of cells based on V_O_-HEO and V_O_-CeO_2_. (b) Rate performance and (c) Galvanostatic charge-discharge profiles of cells based on V_O_-HEO and V_O_-CeO_2_. Voltage profiles of (d) discharge and (e) charge after magnification of V_O_-CeO_2_ and V_O_-HEO based cells under 0.2C. Cycle stability of cells under (f) 0.2C and (g) 1C. (h) Cycle stability of cells based on V_O_-HEO with high sulfur loading under 0.2C.

The battery performance of LSBs assembled with different catalysts were tested. Electrochemical impedance spectroscopy (EIS) was used to further analyze the kinetic behavior of V_O_-HEO and V_O_-CeO_2_ based cells (Fig. 6a). A Nyquist plot consists of a semicircle and a straight line. The fitted curve and corresponding equivalent circuit can be obtained through fitting (Fig. S15). V_O_-HEO based cells have a smaller R_CT_ (31.8 Ω) than V_O_-CeO_2_ based cells (66.7 Ω), indicating a faster charge transfer. Rate performance under different current densities shows that the discharge specific capacities of V_O_-HEO based cells are 1301 mAh g^−1^, 1156 mAh g^−1^, 1062 mAh g^−1^, 954 mAh g^−1^ and 779 mAh g^−1^ at current densities of 0.2C, 0.5C, 1C, 2C and 4C, respectively (Fig. 6b). The specific capacity of 1267 mAh g^−1^ is still being maintained after returning to 0.2C. In contrast, V_O_-CeO_2_ based cells show inferior rate performance. The specific capacity is difficult to recover after returning to 0.2C. The cells show typical charge/discharge curves of LSBs, and the charge and discharge platforms are clearly visible in the galvanostatic charge/discharge curves of V_O_-HEO and V_O_-CeO_2_ based cells (Fig. 6c). The voltage difference between charging and discharging platforms (ΔE) reflects polarization voltage. V_O_-HEO based cells have a lower polarization voltage (ΔE_1 _= 0.1415 V) compared with V_O_-CeO_2_ (ΔE_2 _= 0.1520 V), indicating that the former has faster reaction kinetics. The key behavior of LiPSs during the reaction of LSBs can be further analyzed by enlarging the charge-discharge curve (Fig. 6d and e). The voltage peak appearing at the beginning of the platform during discharge is the process of Li_2_S nucleation. V_O_-HEO based cells have a smaller voltage of nucleation (2.109 V) and a smaller barrier of nucleation (16.7 mV). The voltage peak appearing at the beginning of the platform during charge is the process of Li_2_S decomposition. V_O_-HEO based cells have a smaller voltage of Li_2_S decomposition (2.237 V) and a smaller barrier of Li_2_S decomposition (17.7 mV). These results are consistent with the experimental results of Li_2_S nucleation and decomposition. The ratio between discharge capacities at the high voltage plateau (Q1) and low voltage plateau (Q2) reflects the conversion efficiency of LiPSs (Fig. S16). V_O_-HEO based cells have a higher conversion efficiency for LiPSs (Q2/Q1 = 2.52) compared with V_O_-CeO_2_ (Q2/Q1 = 2.30).

The effects of catalytic stability on cycle performance were investigated. After 150 cycles at 0.2C, V_O_-HEO based cells still maintain a higher specific capacity (990 mAh g^−1^) compared to V_O_-CeO_2_ (773 mAh g^−1^) (Fig. 6f). A long cycle test at 1C was performed for further study (Fig. 6g). V_O_-HEO based cells still maintain the specific capacity of 354 mAh g^−1^ after cycling with a low average capacity fading of 0.032% per cycle. In contrast, V_O_-CeO_2_ based cells only maintain the specific capacity of 163 mAh g^−1^ with an average capacity fading of 0.041% per cycle. V_O_-CeO_2_ based cells have a rapid capacity decay after the first 100 cycles. According to the previous analysis, the unstable V_O_ on the surface of V_O_-CeO_2_ diffuse inward and the surface-active sites are lost during cycling. The reduced catalytic performance eventually leads to the attenuation of battery cycle performance. Due to the high stability of the V_O_ on V_O_-HEO, the cells maintain high catalytic performance during long cycling, resulting in better cycle performance.

LSBs with high sulfur loads were tested to explore the possibilities of practical application. V_O_-HEO based cells with high sulfur loading of 5.1 mg cm^−2^ and 6.4 mg cm^−2^ exhibit specific capacities of 1305 mAh g^−1^ and 1185 mAh g^−1^, respectively, corresponding to the areal capacity of 6.65 mAh cm^−2^ and 7.58 mAh cm^−2^ (Fig. S17). Meanwhile, the cells with a high sulfur areal loading of 5.1 mg cm^−2^ maintain a specific capacity of 766 mAh g^−1^ after 50 cycles under 0.2C (Fig. 6h). The lean electrolyte setup and thin Li foil are used to eliminate the influence of excessive electrolytes and excessive lithium anodes (Fig. S18). Under the conditions of 4.78 mg cm^−2^ and a low E/S ratio of 10, V_O_-HEO based cells exhibit a higher discharge specific capacity (833 mAh g^−1^) than V_O_-CeO_2_ based cells (703 mAh g^−1^). After 100 cycles, V_O_-HEO based cells maintain a higher capacity retention rate (75.5%) compared with V_O_-CeO_2_ based cells (67.3%). Besides, the performance comparison with previous catalysts in LSBs in Table S2 highlights the advantages of V_O_-HEO.

## CONCLUSION

In summary, we obtained intrinsically stable and active V_O_ in catalysts *via* a high-entropy strategy for high performance lithium-sulfur batteries. The 2D V_O_-HEO was successfully obtained by simple template synthesis. Experiments and DFT calculations confirm that the complex structure formed by five different metals plays an important role in improving catalytic performance. Specifically, (1) the unique electronic and crystal structure of HEO alters its intrinsic properties, leading to varied formation energies and a higher energy barrier for V_O_ diffusion, which prevents inward migration of surface V_O_, improving its stability. (2) The multiple metal atoms exposed on the surface of V_O_-HEO provide more adsorption sites, strengthening the anchoring of LiPSs and suppressing the shuttle effect. (3) These exposed metal atoms also weaken the chemical bonds in LiPSs and lower the associated energy barrier, accelerating the reaction kinetics of LiPSs conversion. The cells based on V_O_-HEO deliver a specific capacity as high as 1301 mAh g^−1^ under 0.2C and a low average capacity fading of 0.032% per cycle after 2000 cycles under 1C. Besides, an areal capacity of 7.58 mAh cm^−2^ is exhibited under 0.2C with a high sulfur areal loading of 6.4 mg cm^−2^. The results show that high entropy is an effective way to keep V_O_ both active and stable. It overcomes the common challenge where traditional defective catalysts struggle to achieve both, offering inspiration for advancing the use of high-entropy materials in broader catalytic applications.

## METHODS

The detailed experimental reagents and methods can be found in the online Supplementary data.

## Supplementary Material

nwaf375_Supplemental_File
