# Supplementary material for: Intrinsic stabilization of vacancies in catalysts via high-entropy approach for lithium-sulfur batteries
Source: Natl Sci Rev. 2025 Sep 8;12(10):nwaf375. doi: 10.1093/nsr/nwaf375 (PMC12499420; doi:10.1093/nsr/nwaf375)
Supplement: nwaf375_Supplemental_File [file nwaf375_supplemental_file.pdf]

1. Experimental Procedures

1.1 Synthesis of V_O-HEO and V_O-CeO_2

4g PVP was slowly added into 20ml water until completely dissolved into a transparent solution under vigorous stirring. 0.462 g of $Cu(NO_3)_2$, 0.366 g of $Zn(NO_3)_2 \cdot 6H_2O$, 0.231 g of $Al(NO_3)_3 \cdot 9H_2O$, 2.675 g of $Ce(NO_3)_3 \cdot 6H_2O$ and 0.264 g of $Zr(NO_3)_4 \cdot 5H_2O$ were added into the above solution under vigorous stirring for 30 min, in which the PVP/metal salts weight ratio was 1. The solution was quickly cooled directly through liquid nitrogen and then freeze-dried for 48h. The obtained powders were placed in a crucible and heated to 450 ℃ at 1℃/min for 6 h in air. Finally, the naturally cooled powders were heated to 450 ℃ at 5℃/min for 2 h in H_2/Ar atmosphere (5% H_2) to obtain V_O-CeO_2. Only $Ce(NO_3)_3 \cdot 6H_2O$ was added to obtain V_O-CeO_2 without changing other conditions, in which the PVP/metal salts weight ratio was 1.

PVP, $Cu(NO_3)_2$, $Zn(NO_3)_2 \cdot 6H_2O$, $Ce(NO_3)_3 \cdot 6H_2O$ and $Zr(NO_3)_4 \cdot 5H_2O$ were purchased from Shanghai Aladdin Biochemical Technology Co., Ltd. $Al(NO_3)_3 \cdot 9H_2O$ was purchased from Shanghai Bide Pharmatech Co., Ltd. 5% H_2/Ar atmosphere was purchased from Yingkou Jiahe Gas Co., Ltd.

1.2 Preparation of V_O-HEO and V_O-CeO_2 separators

Firstly, 4 mg of the V_O-HEO or V_O-CeO_2 powders obtained in the above experiments were placed in two glass bottles. Subsequently, Super-P and a 5 *wt.*% PVDF solution dissolved in N-methylpyrrolidone (NMP) were added into the above glass bottles (catalysts:Super-P: PVDF = 8:1:1). Then, 12 mg NMP was added into the above glass bottles and the bottles were put into an ultrasonic machine for ultrasonic mixing. The PVDF and the powders were adhered together uniformly during this process. Finally, the dispersed mixture was coated onto the Celgard polypropylene (PP) separators separately by vacuum filtration, and then dried at 60 ℃ for 12 h to obtain the separators coated with V_O-HEO and V_O-CeO_2.

1.3 Assembly and electrochemical measurements of Li_2S_6 symmetric cells

S and Li_2S with a ratio of 5:1were placed in a solution of DOL/DME (V_{DOL}/V_{DME}=1:1) with 1 mol/L LiTFSI and 0.2 mol/L $LiNO_3$, and stirred at 40 ℃ until completely dissolved to obtain Li_2S_6 solution. The V_O-HEO or V_O-CeO_2 powders, and PVDF binder (5 *wt.*% with NMP) were mixed in a mixer at a ratio of 9:1 with appropriate amount of NMP to obtain a uniform slurry. Whereafter, the prepared slurry was coated evenly on the aluminum foil current collector using a mold and dried at room temperature for 12 h, and a disk with a diameter of 12 mm was punched to prepare electrodes. Cyclic voltammetry measures were performed at the scan rate of 2 mV s^{-1} between 1 V and -1 V. The symmetric cells were assembled for testing using the punched electrodes as both the cathodes and anodes, with Li_2S_6 solution serving as the electrolyte.

1.4 Experiments on Li_2S nucleation and decomposition

Similar to the preparation method of Li_2S_6, S and Li_2S with a ratio of 7:1were placed in a mixed solution of DOL/DME (V_{DOL}/V_{DME}=1:1) with 1 mol/L LiTFSI and 0.2 mol/L $LiNO_3$ and stirred at 40 ℃ until completely dissolved to obtain Li_2S_8 solution. The Celgard 2400 separator was sandwiched between V_O-HEO and V_O-CeO_2 sulfur-free cathode and lithium metal anode, and Li_2S_8 solution was added as the electrolyte to assemble batteries for experiments on Li_2S nucleation and decomposition. For the Li_2S nucleation experiment, the battery obtained above was discharged galvanostatically to 2.13 V under 0.1 mA, and then discharged potentiostatically at 2.05 V until the discharge current dropped to 10^{-5} A to collect the full charge and evaluate the Li_2S nucleation/growth rate. For the Li_2S decomposition experiment, the battery was discharged galvanostatically to 1.8 V under 0.1 mA, and then discharged galvanostatically to 1.7 V under 0.01 mA to ensure completely conversion to Li_2S. After complete conversion, potentiostatically charging was performed at a voltage of 2.4 V until the charging current was below 10^{-5} A to achieve complete decomposition of Li_2S.

1.5 Assembly and electrochemical measurements of lithium-sulfur batteries

Ketjen black carbon and sulfur powder were mixed (C:S = 7:3) by grounding for about 1 hour in a mortar, and then the mixed powder was placed in a Teflon-lined

autoclave for 12 h at 155 ℃ followed with melting-diffusion. The autoclave cooled down to room temperature naturally to obtain a carbon-sulfur composite with a sulfur content of 70 *wt*.%. The carbon-sulfur composite and LA133 binder (5 *wt*.% with water) were mixed in a mixer with a ratio of 9:1 with appropriate amount of water as a solvent to obtain a uniform slurry. Whereafter, the prepared slurry was spread evenly on the carbon-coated aluminum foil current collector using a mold and dried at room temperature for 12 h, and the coated aluminum foil was punched into disks with a diameter of 12 mm and used as cathodes with a sulfur load of approximately 0.8 mg cm^{-2}. The carbon-sulfur electrode as cathode, lithium metal as anode, PP separator coated with functional materials, and 30 μL of electrolyte were assembled to obtain standard 2025 button lithium-sulfur batteries. The electrolyte was composed of 1 mol L^{-1} lithium bis(trifluoromethylsulfonyl)imide (LiTFSI) and 0.2mol/L $LiNO_3$ dissolved in a mixed solvent of 1, 3-dioxolane (DOL) and dimethoxymethane (DME) (V_{DOL}/V_{DME}=1:1). Cyclic voltammetry (CV) and electrochemical impedance spectroscopy (EIS) were measured on an electrochemical workstation (ChenHua CHI650D). The lower and upper voltage limits for cyclic voltammetry were 1.7 V and 2.8 V respectively, and the frequency range for electrochemical impedance spectroscopy was 1 MHz to 0.1 mHz with an amplitude of 5 mV. The charge/discharge tests and cycling tests were measured from 1.7 V to 2.8 V by using a test system of batteries (Neware).

1.6 Material characterization

X-ray diffractometer with Cu Kα radiation (XRD, PANalytical X'Pert PRO, 40 mA, 40 kV) was used to characterize the crystalline structure of the prepared materials. Surface elements were analyzed by X-ray photoelectron spectroscopy measurements (XPS, Thermo Scientific). The morphology was characterized by transmission electron microscopy (TEM, FEI Tecnai G2 F30) and scanning electron microscopy (SEM, Hitachi SU8010). The contents of each substance in the composites were measured via a thermogravimetric analyzer system (Linseis STA PT 1600). The ultraviolet-visible (UV-vis) spectrum analysis was characterized via a Shimadzu UV-2450 Spectrophotometer. The Brunauer-Emmett-Teller method (BET, ASAP 2020,

Micromeritics) was used to characterize the specific surface areas of the prepared materials. Special aberration corrected scanning transmission electron microscope, (AC-STEM, FEI Titan G2 60-300) was used to characterize the atomic configuration of the prepared materials. Electron paramagnetic resonance (EPR, ELEXSYS-II E500 CW-EPR) was used to characterize the oxygen vacancy concentration.

1.7 Theoretical calculations

All ab initio calculations of this manuscript were carried out using the Quantum ESPRESSO package by a method of the density-functional theory (DFT), and the generalized gradient approximation (GGA) in the Perdew-Burke-Ernzerhof (PBE) formulation was used. The ultrasoft pseudopotentials were chosen to describe the core electrons. The dispersion corrections were introduced by a method of DFT-D3. The energy cutoff of electronic wave functions and the charge density were set to 35 and 280 Ry, respectively. The CeO_2 (111) surface was modeled using a (3×3) supercell slab and a 15 Å vacuum layer was modeled on the slab. 22.22%, 11.11%, 5.56% and 5.56% of Ce were replaced with Cu, Zn, Al and Zr respectively to obtain the HEO surface. Part of the O atoms were removed to form oxygen vacancies. The Brillouin zone with a 2 × 2 × 1 k-point grid was sampled. The convergence thresholds of total energy and force threshold were set to 10^{-6} Ry and of 10^{-3} Ry/Bohr for structural optimization, respectively. $\Delta E = E_{slab+ads} - (E_{slab} + E_{ads})$ was used for calculating the binding energy between substrate and adsorbed sulfur species. Among them, $E_{slab+ads}$ is the total energy of all absorbed system, E_{ads} represents the energy of the absorbed sulfur species and E_{slab} represents the energy of the slab after absorbing. $\sigma = \sigma^{unrel} + E^{rel}$ and $\sigma^{unrel} = 1/2(E_{surf} - N_{atoms} \bullet E_{bulk})$ are used for calculating the surface energies. Among them, σ is the surface energy, E^{rel} represents the relaxation energy of the slab, E_{bulk} represents the energy of single atom in bulk, E_{suf} represents the energy of the freshly cut slab, N_{atoms} is the number of the atoms in slab. $E_{Vo} = E_{defect} - E_{perfect} + 1/2\mu_{O2}$ is used for calculating the formation energies of oxygen vacancies. Among them, E_{Vo} represents the formation energies of oxygen vacancies, E_{defect} represents the energy of slab with oxygen vacancies, $E_{perfect}$ represents the energy of slab without oxygen vacancies and μ_{O2} is the chemical potential of O_2. $\Delta G = \Delta E + \Delta ZPE - T\Delta S$ was used

for calculating Gibbs free energy change (ΔG) of each sulfur reduction step. Among them, ΔE is the total energy difference, ΔZPE represents the change of zero-point energy, T was set as 298.15 K and ΔS is the change of entropy, respectively. The climbing image nudged elastic band (CI-NEB) method was used to simulate Li_2S decomposition possess ($Li_2S \rightarrow Li^+ + e^- + LiS$).

1.8 Assembly and electrochemical measurements of *in situ* Raman batteries

The synthesis of the cathode material was prepared by the same method with the lithium-sulfur batteries. The slurry was coated on carbon-coated aluminum foil. After drying at 60 °C for 12 h, the coated aluminum foil was cut into a disk with a diameter of 12 mm and used as cathode. *In situ* Raman battery jar purchased from Beijing Scistar Technology Co.Ltd was used to assemble batteries. The cathode, lithium metal as anode, PP separator, and 45 μL of electrolyte were assembled to obtain *in situ* Raman batteries. *In situ* Raman spectra were tested on a Lab RAM HR800 Raman spectrometer.The wavelength of the incident laser was 532 nm.

2. Supporting Figures

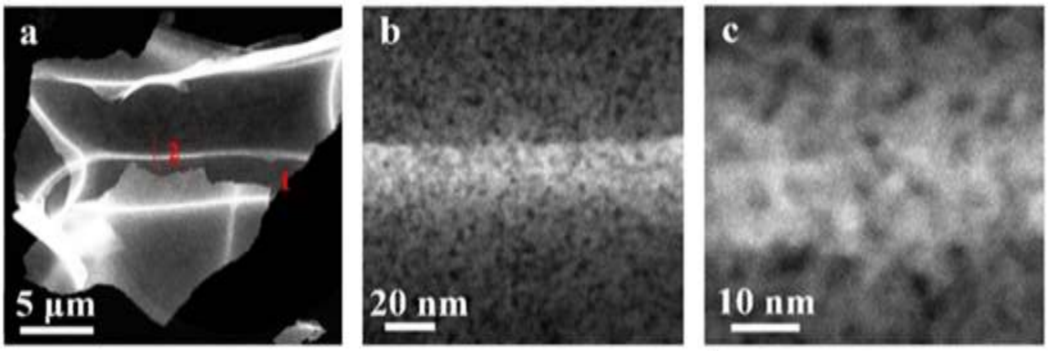

Figure S1. AC-STEM images of V_O-HEO in different magnifications

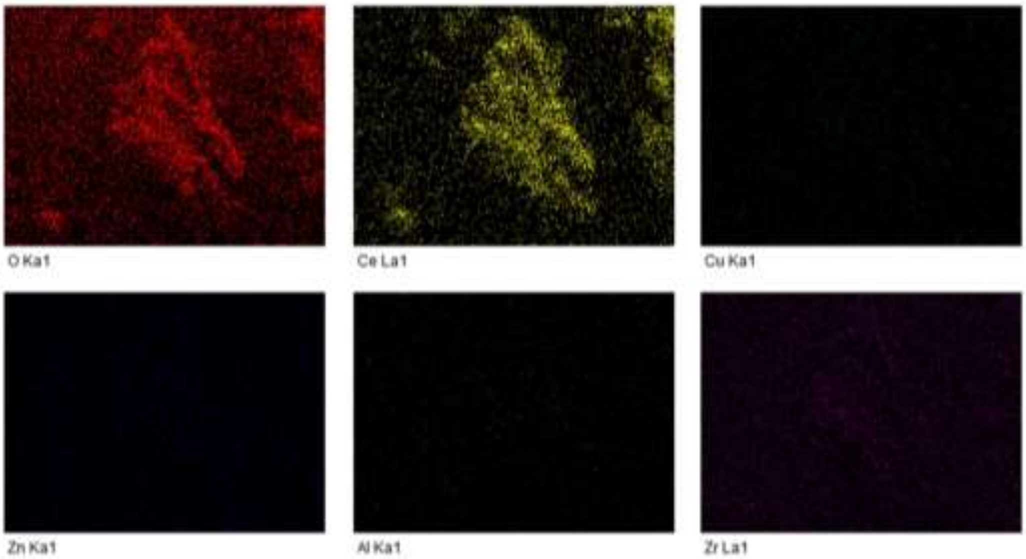

Figure S2. Elemental mapping images of V_O-CeO_2

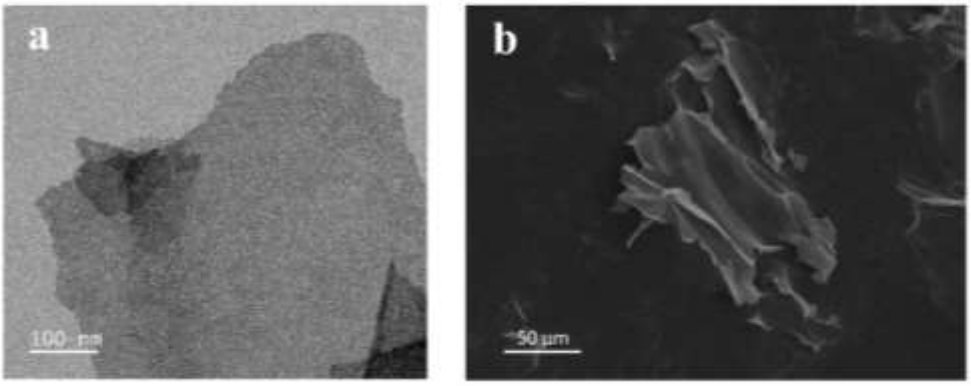

Figure S3. (a) V_O-HEO and (b) V_O-CeO_2 origin images of elemental mapping

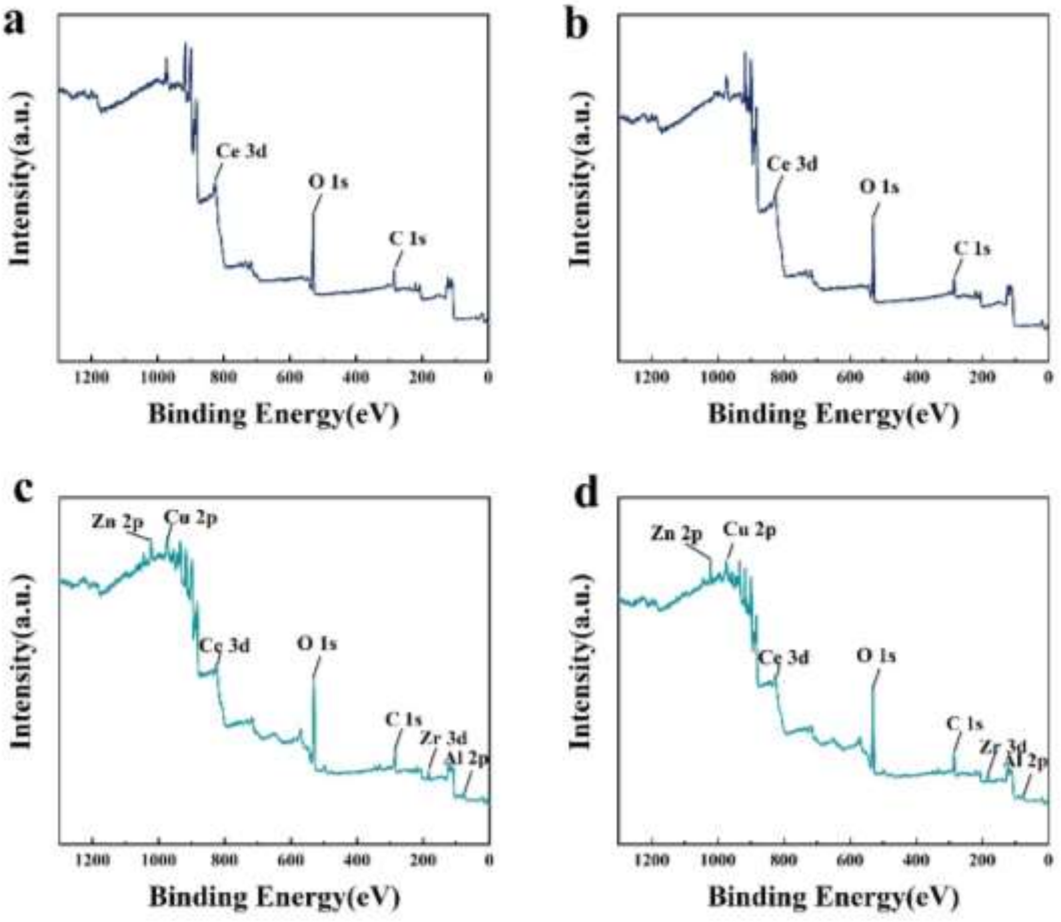

Figure S4 The full XPS spectra of (a) V_O-CeO_2, (b) V_O-CeO_2 before cycling, (c) V_O-HEO and (d) V_O-HEO after cycling.

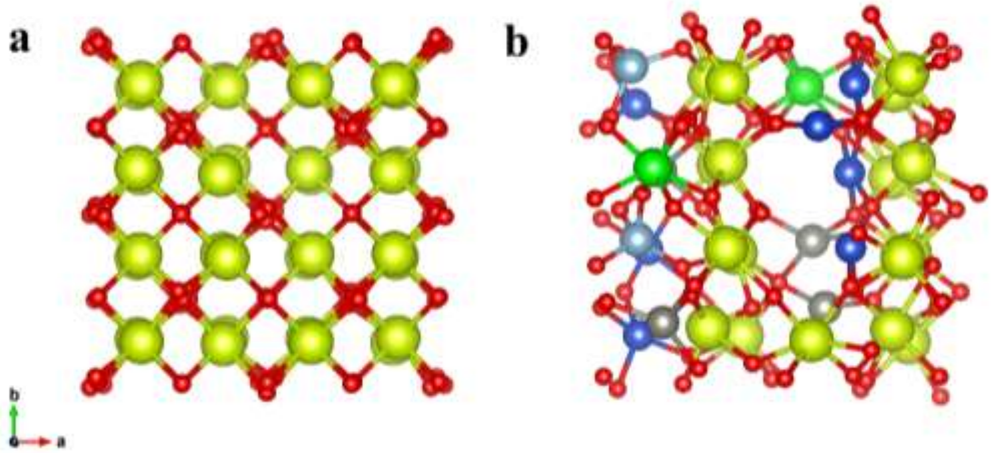

Figure S5. Optimized geometries of (a) V_O-CeO_2 and (b) V_O-HEO

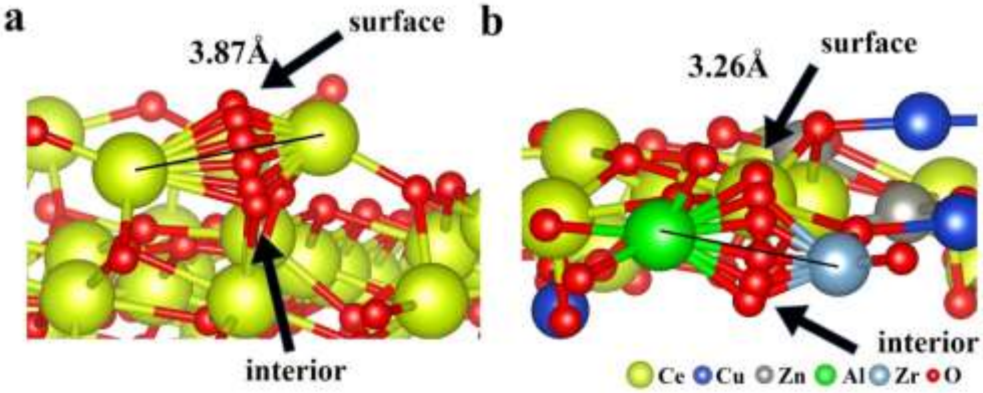

Figure S6. The diffusion pathways of V_O in (a) V_O-CeO_2 and (b) V_O-HEO

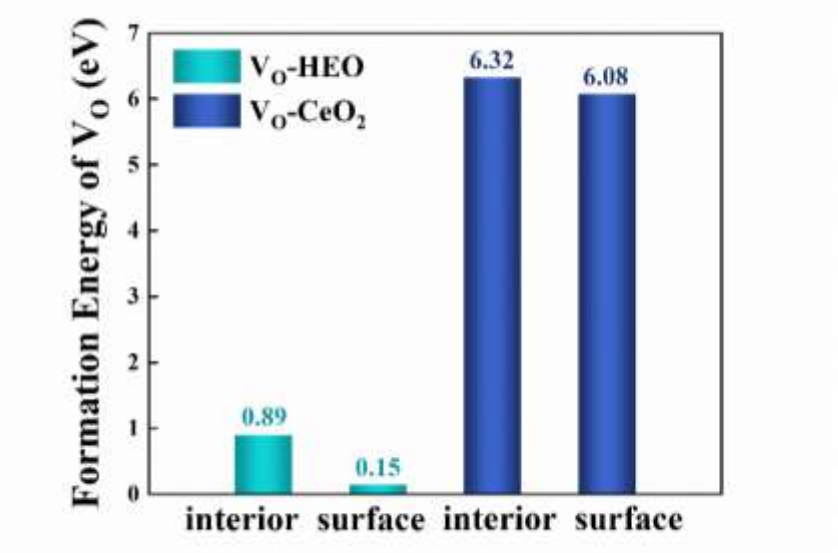

Figure S7. Formation energies of V_O in surface and interior of V_O-HEO and V_O-CeO_2.

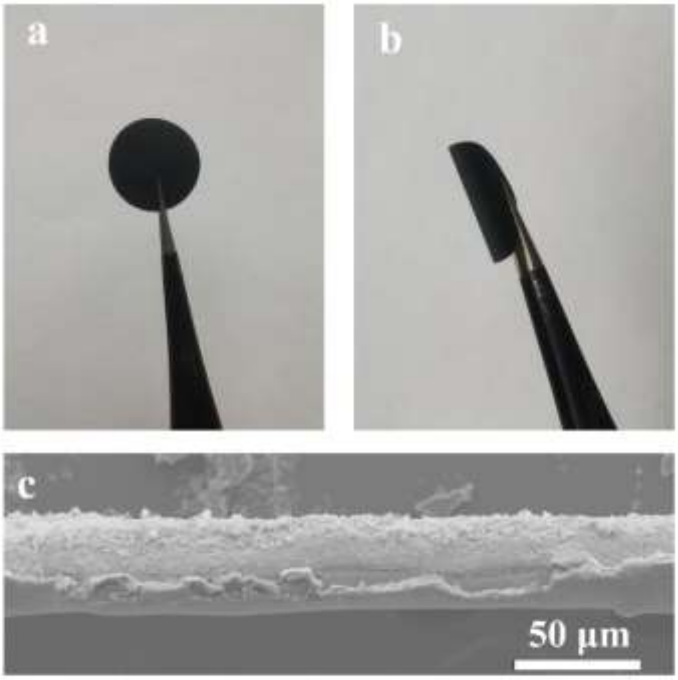

Figure S8. Digital photos of (a) V_O-HEO separator and (b) V_O-HEO separator after bending. (c) Cross-section SEM image of V_O-HEO separator.

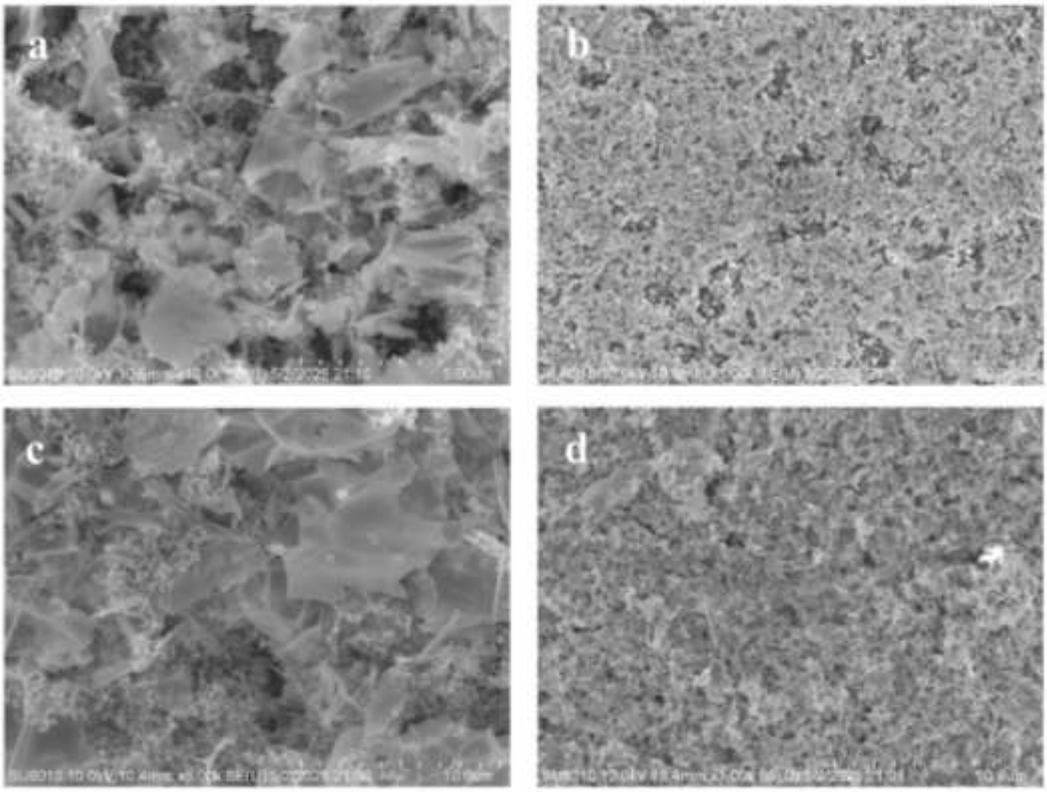

Figure S9. SEM images of (a-b) V_O-HEO separator and (c-d) V_O-CeO_2 separator**.**

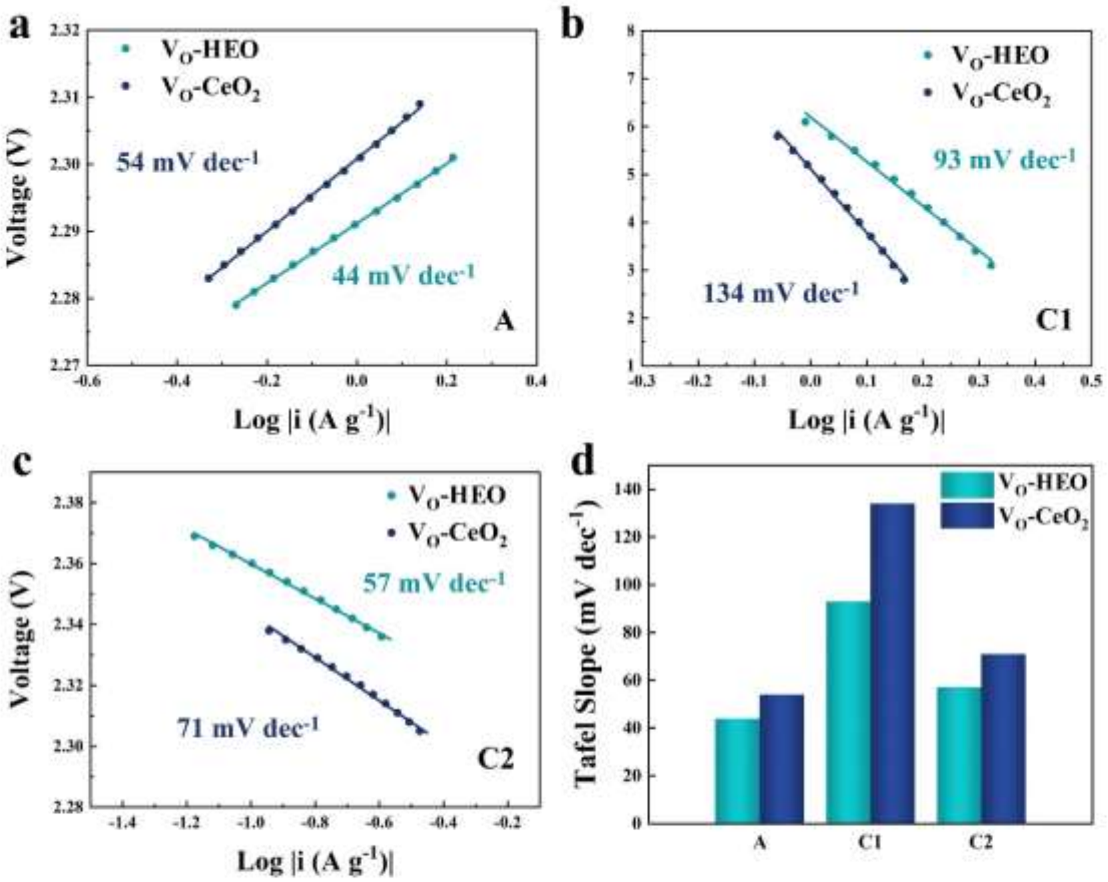

Figure S10. (a-c) Tafel slopes and (d) statistical plot of slopes of V_O-HEO and V_O-CeO_2 based cells

Table S1. Different polysulfides and their corresponding characteristic peak

Polysulfides	Characteristic Peaks
Li_2S_8	156, 217 and 475 cm^{-1}
Li_2S_6	393 cm^{-1}
Li_2S_4	201 cm^{-1}
Li_2S_4 and Li_2S_2	459 cm^{-1}

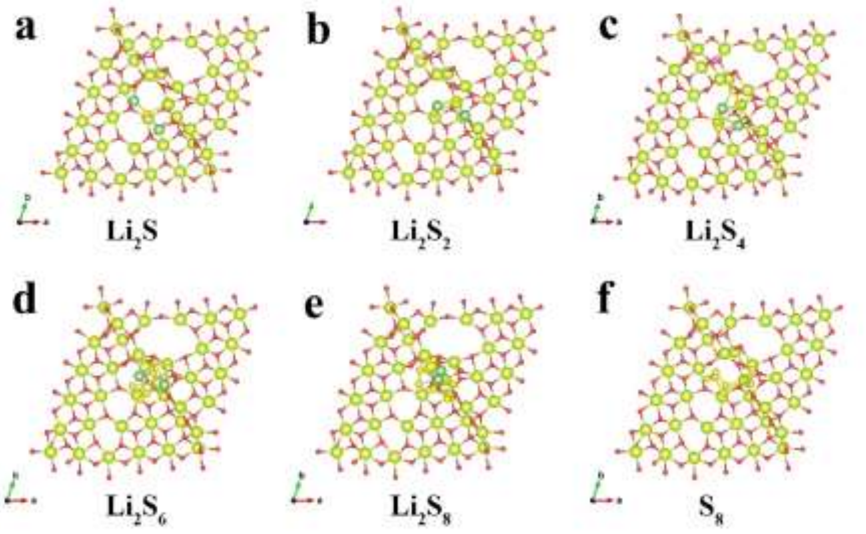

Figure S11. The optimized geometries of different sulfur species on V_O-HEO during reduction processes.

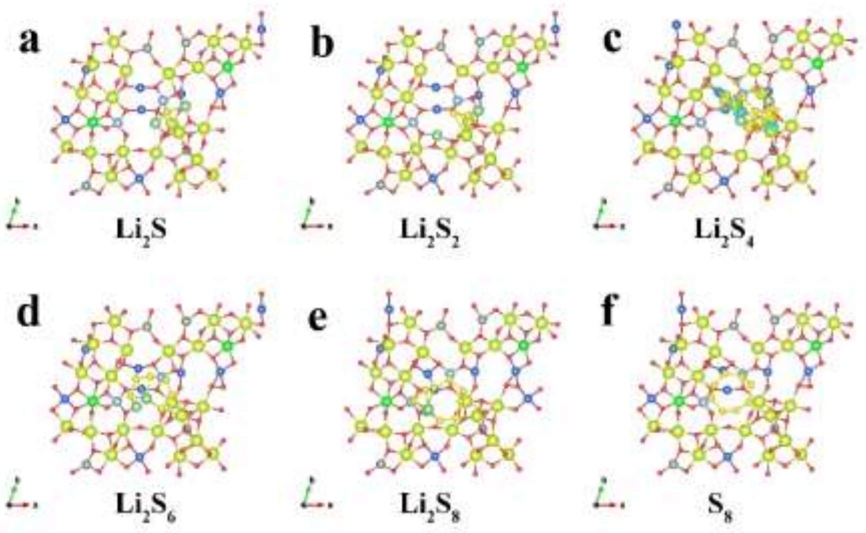

Figure S12. The optimized geometries of different sulfur species on V_O-CeO_2 during reduction processes.

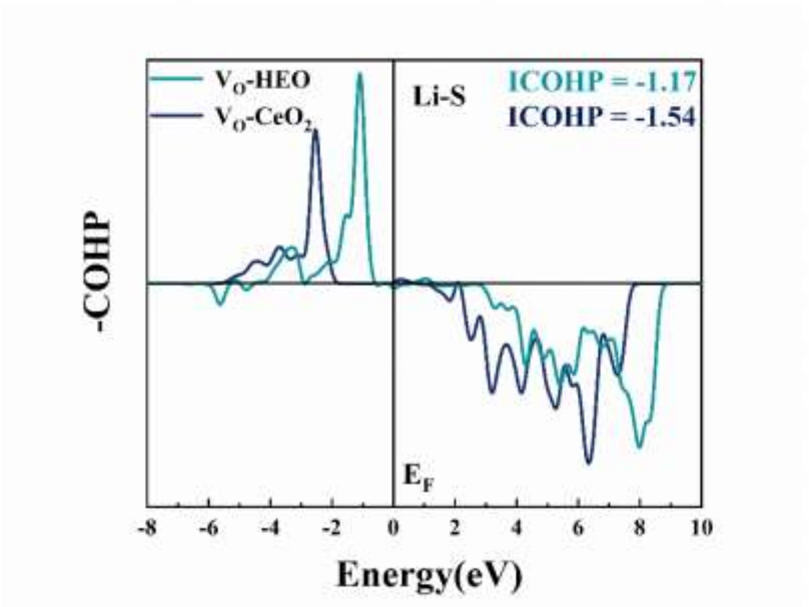

Figure S13. COHPs of Li-S bond adsorbed on V_O-HEO and V_O-CeO_2.

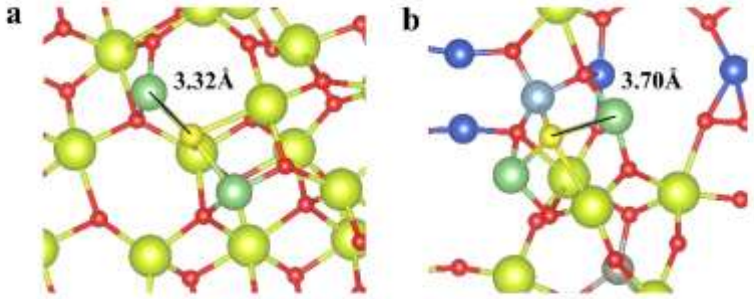

Figure S14. Optimized geometries of Li_2S adsorbed on (a) V_O-CeO_2 and (b) V_O-HEO.

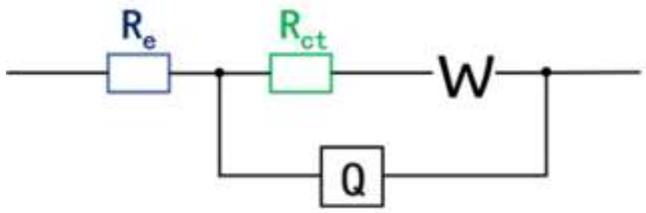

Figure S15. Corresponding equivalent circuits of Li-S cells

R_e: The internal resistance of the electrolyte;
R_{ct}: The charge-transfer resistance, related to the electrode reaction kinetics;
Q: Capacitance of the electrode bulk in high-frequency region considering the dispersion effect;
W: The semi-infinite Warburg diffusion impedance.

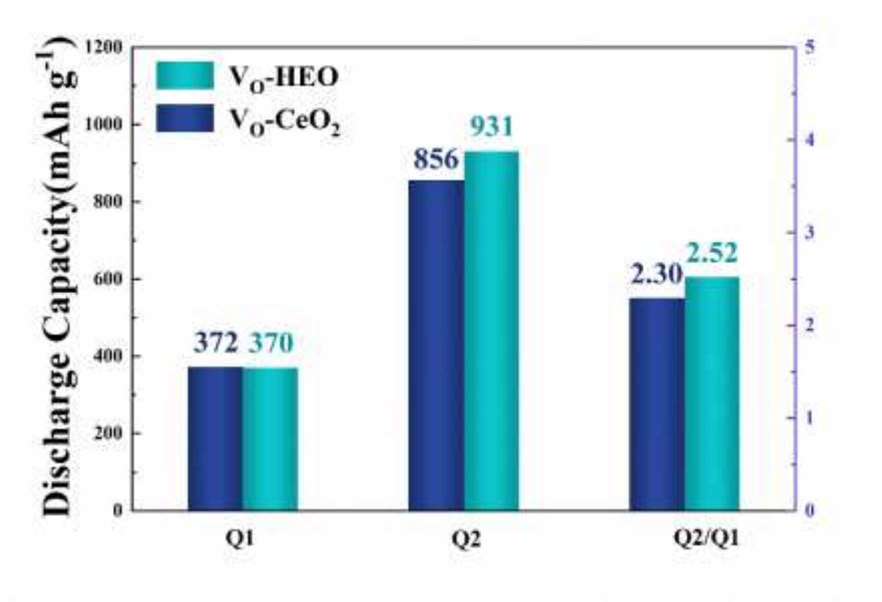

Figure S16. Comparison of discharge capacity at different voltage plateau

Table S2 Performance comparison between V_O-HEO and previously catalysts

Materials	Initial Capacity (mAh g^{-1})	Rate	Cycle Number	Rate	Capacity Decay Rate per Cycle	Ref
V_O-HEO	1301	0.2 C	2000	1 C	0.032%	This work
HSCo/TiO_{2-x}	1177	0.2 C	500	1 C	0.06%	Adv. Mater. 2025, 2502075[1]
CNT@f-CoNC	1223	0.2 C	300	1 C	0.033%	Angew. Chem. Int. Ed. 2025[2]
Zn-COF	1305	0.2 C	500	1 C	0.05%	Adv. Funct. Mater. 2025[3]
P-In_2S_{3-x}	1016	0.2 C	1000	1 C	0.045%	Nano Energy 2025[4]
Al_2O_3@mG	1307	0.2 C	1600	1 C	0.032%	EnergyEnviron. Sci., 2025[5]
$CoTe_2$/Co-O-NC	1210	0.2 C	500	0.5 C	0.046%	Adv. Funct. Mater. 2025[6]
HEA	948	0.2 C	500	1 C	0.06%	Adv. Sci.2024[7]
HE-MXene	1239	0.2 C	400	1 C	0.057%	EnergyEnviron. Sci., 2024[8]
Co_7Fe_3Co	1125	0.1 C	1000	1 C	0.046%	Adv. Funct. Mater. 2025[9]
Co-VC	1212	0.2 C	700	1 C	0.036%	Adv. Funct. Mater. 2024[10]

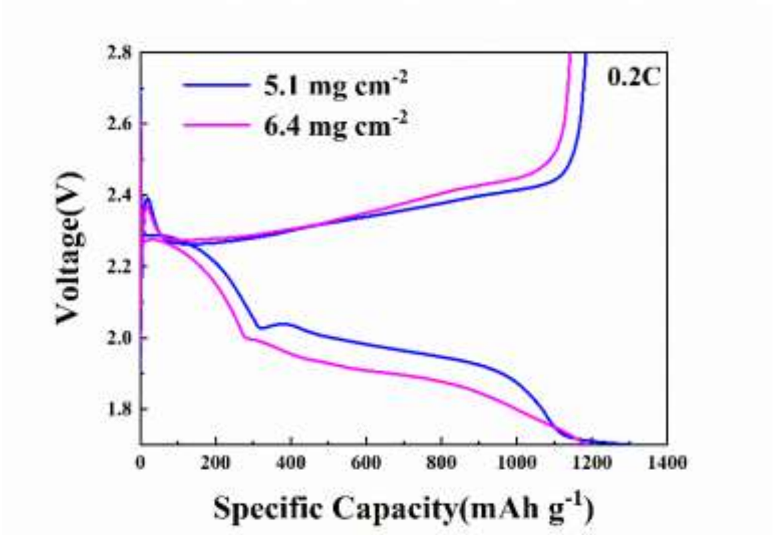

Figure S17. Galvanostatic charge-discharge profile of cells based on V_O-HEO with high sulfur loading under 0.2C.

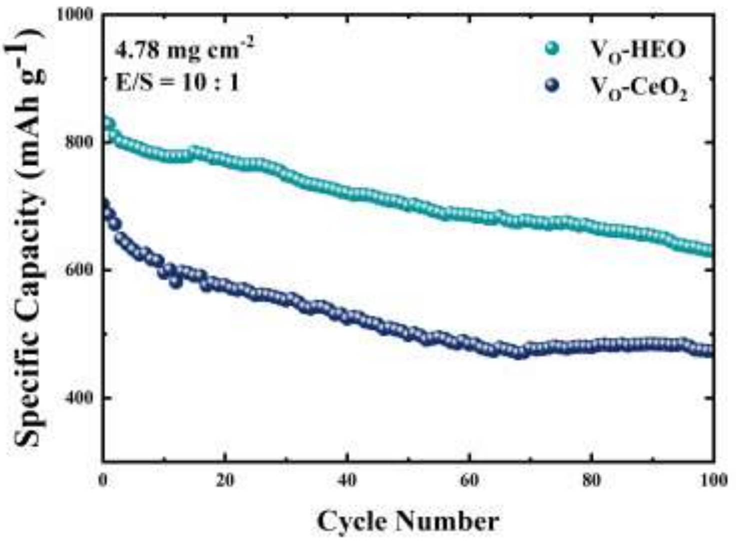

Figure S18. Cycle stability of cells based on V_O-HEO and V_O-CeO_2 with high sulfur loading and low E/S under 0.2C.

References

1. Wang J, Zhang X, Liu J *et al.* High-Spin Cobalt Enables Strong Metal-Sulfur Orbital Hybridization for Accelerated Polysulfide Conversion in Lithium-Sulfur Batteries. *Adv Mater* 2025; 2502075.
2. Li M, Liu H, Li H *et al.* Electron-Deficient Cobalt Centers Realized by Rational p─π Conjugation Regulation for High-Performance Li─S Batteries. *Angew Chem Int Ed* 2025; **64**: e202503174.
3. Yang K, Zhao F, Li C *et al.* Developing a One-Pot Strategy to Synthesize Metal–Covalent Organic Frameworks as Catalysts for Polysulfide Conversion and Ion Calibrators for Lithium

Deposition. *Adv Funct Mater*. 2025; 2501980.
4. Huang Z, Jiao X, Lei J *et al.* Activated d-electrons of p-block metals by reconfigured electron spin for kinetically boosting sulfur conversion of lithium-sulfur batteries. *Nano Energy*. 2025; **139**: 110979.
5. Gu J, Shi Z, Mu Y *et al.* Sustaining vacancy catalysis via conformal graphene overlays boosts practical Li–S batteries. *Energy Environ Sci* 2025; **18**: 5940-5951.
6. Yang Z, Yan R, Han J *et al.* Oxygen Bridges of $CoTe_2$/Co─O─NC Enhancing Adsorption-Catalysis of Polysulfide for Stable Lithium–Sulfur Batteries. *Adv Funct Mater*. 2025; **35**: 2417834.
7. Xu Y, Yuan W, Geng C *et al.* High-Entropy Catalysis Accelerating Stepwise Sulfur Redox Reactions for Lithium–Sulfur Batteries. *Adv Sci* 2024; **11**: 2402497.
8. Xu M, Zhu Q, Li Y *et al.* Atom-dominated relay catalysis of high-entropy MXene promotes cascade polysulfide conversion for lithium–sulfur batteries. *Energy Environ Sci* 2024; **17**: 7735-7748.
9. Sun L, Xu H, Xie J *et al.* D-Band Center Modulation of Metallic Co-Incorporated Co_7Fe_3 Alloy Heterostructure for Regulating Polysulfides in Highly Efficient Lithium-Sulfur Batteries. *Adv Funct Mater* 2025; **35**: 2416826.
10. Song Y, Sun Y, Chen L *et al.* Seeding Co Atoms on Size Effect-Enabled V_2C MXene for Kinetically Boosted Lithium–Sulfur Batteries. *Adv Funct Mater* 2024; **34**: 2409748.
